# The use of coproduction to inform an evidence-based service delivery model for mental health service users with complex needs

**DOI:** 10.1192/bjo.2021.761

**Published:** 2021-06-18

**Authors:** Pooja Saini, Rajan Nathan, Laura Sambrook, Sam Burton, Hana Roks, Anna Balmer, Jason McIntyre, Antony Martin, Amrith Shetty

**Affiliations:** 1 Liverpool John Moores University; 2 Cheshire and Wirral Partnership NHS Foundation Trust; 3 QC Medica

## Abstract

**Aims:**

Co-production recognises that people who use social care services (and their families) and third sector organisations within community settings have knowledge and experience that can be used to help make services better for services users and those who care for them. This study shares the coproduction that took place in the design of a mixed methods study that aims to understand: the profile and history of service users currently defined as having complex needs; the decision-making processes by clinicians that lead to these individuals entering this complex group; service users and carers experience of service use; and, the associated costs. This study involves a comprehensive evaluation that aims to inform an evidence-based service delivery model for mental health service users with complex needs.

**Method:**

A study stakeholder group, including clinicians, academics, service users, housing associations, health economists, and statisticians was formed from the outset to inform the mixed methods design, combining quantitative (in-depth analysis of patient records and economic evaluation) and qualitative (written medical notes and in-depth interviews with service users, carers, and clinicians) methods. The study included five components: (1) a quantitative description and analysis of the demographic clinical characteristics of the patient group; (2) an economic evaluation of direct medical costs, direct non-medical costs, and indirect costs for each patient; (3) semi-structured interviews about patients and carers experiences; (4) data from components 1-3 was used to co-produce vignettes jointly with the stakeholders group; and, (5) semi-structured interviews about clinical decision-making by clinicians in relation to this patient group by using the vignettes as example case studies.

**Result:**

Coproduction took place at each stage of the study, including the design, development of data collection tools, data analysis and formation of the vignettes required for stage five. The results demonstrated how co-production and multiagency working have been evident throughout the process of designing the study, the continuous engagement throughout the analysis, dissemination and implementation of the findings.

**Conclusion:**

The findings support the application of the core principles of co-production in the design, set-up and implementation of research within an NHS Trust as demonstrable by the acceptability and collaborative working within the study. The study's key outcomes were to: examine the resource use and cost impact associated with alternative care pathways to the NHS and other sectors of the economy (including social care); explore patient health and non-health outcomes associated with alternative care pathways; and, gain an understanding of a complex service user group and how decisions are made in their treatment to inform how services are delivered in the future and made more person-centred and consistent.

